# Associations between patient safety culture and workplace safety culture in hospital settings

**DOI:** 10.1186/s12913-024-10984-3

**Published:** 2024-05-02

**Authors:** Brandon Hesgrove, Katarzyna Zebrak, Naomi Yount, Joann Sorra, Caren Ginsberg

**Affiliations:** 1 Westat, Rockville, MD USA; 2 Agency for Healthcare Research and Quality, Rockville, MD USA

**Keywords:** Healthcare, Health care, Workplace safety, Patient safety, Workforce safety, Safety culture, Organizational culture, Survey

## Abstract

**Background:**

Strong cultures of workplace safety and patient safety are both critical for advancing safety in healthcare and eliminating harm to both the healthcare workforce and patients. However, there is currently minimal published empirical evidence about the relationship between the perceptions of providers and staff on workplace safety culture and patient safety culture.

**Methods:**

This study examined cross-sectional relationships between the core Surveys on Patient Safety Culture™ (SOPS®) Hospital Survey 2.0 patient safety culture measures and supplemental workplace safety culture measures. We used data from a pilot test in 2021 of the Workplace Safety Supplemental Item Set, which consisted of 6,684 respondents from 28 hospitals in 16 states. We performed multiple regressions to examine the relationships between the 11 patient safety culture measures and the 10 workplace safety culture measures.

**Results:**

Sixty-nine (69) of 110 associations were statistically significant (mean standardized β = 0.5; 0.58 < standardized β < 0.95). The largest number of associations for the workplace safety culture measures with the patient safety culture measures were: (1) overall support from hospital leaders to ensure workplace safety; (2) being able to report workplace safety problems without negative consequences; and, (3) overall rating on workplace safety. The two associations with the strongest magnitude were between the overall rating on workplace safety and hospital management support for patient safety (standardized β = 0.95) and hospital management support for workplace safety and hospital management support for patient safety (standardized β = 0.93).

**Conclusions:**

Study results provide evidence that workplace safety culture and patient safety culture are fundamentally linked and both are vital to a strong and healthy culture of safety.

**Supplementary Information:**

The online version contains supplementary material available at 10.1186/s12913-024-10984-3.

## Background

About 10% of patients internationally have adverse events^1^ in hospitals, and about half of these adverse events are considered to be preventable [1, 2]. About 7% of these adverse events result in death and about half result in temporary or permanent disability. As discussed in the seminal publication *To err is human*, building a culture of safety is a key component of preventing medical errors and harm to patients [3]. A growing body of domestic and international research has shown associations between better patient safety culture and reduced adverse events and improved patient experience [4–8].

In 1993, the Health and Safety Commission defined safety culture in the following manner: “The safety culture of an organisation is the product of individual and group values, attitudes, perceptions, competencies, and patterns of behaviour that determine the commitment to, and the style and proficiency of, an organisation’s health and safety management. Organisations with a positive safety culture are characterized by communications founded on mutual trust, by shared perceptions of the importance of safety and by confidence in the efficacy of preventative measures” [9]. Since then, the concept of safety culture has been applied to the healthcare setting, especially hospitals, and it has been demonstrated that the employer’s safety culture influences the attitude and behaviors of both providers and staff, thus contributing to the overall safety of the organization [10]. To comprehensively assess safety culture in the hospital setting, the Agency for Healthcare Research and Quality (AHRQ) sponsored the development of the Surveys on Patient Safety Culture® (SOPS®) Hospital Survey that assesses provider and staff perceptions of the extent to which the organizational culture in hospitals supports patient safety [11].

Although safety culture in healthcare has, until recently, focused on patient safety, several major reports and events, including the World Health Organization’s World Patient Safety Day 2020 [12], the Institute for Healthcare Improvement (IHI) National Steering Committee for Patient Safety’s National Actional Plan to Advance Patient Safety [13], and the National Plan for Health Workforce Well-Being [14], have identified workforce safety as a critical component of advancing patient safety. Workplace safety, including stress and burnout, is a critical issue, as the overexertion injury rate for hospital workers is more than twice the national average of U.S. full time workers [15]. The most important risk factor for these injuries is the manual lifting, moving, and repositioning of patients [16]. Further, these injuries are frequently underreported [17–19]. Additionally, healthcare workers are four times more likely to be victims of verbal and physical workplace violence and aggression than workers in other private industries [20, 21]. The COVID-19 pandemic further exacerbated the safety of healthcare workers through shortages of personal protective equipment, high risk and fears over becoming infected and infecting family members with the virus [22, 23], and increased patient loads and staffing shortages [24, 25].

As a response to this increased concern about the safety of healthcare workers, AHRQ funded the development of the supplemental item set for the SOPS Hospital Survey which focused on the workplace safety of providers and staff in the hospital setting. Recent prominent reports and integrative models of safety culture have shown that not only is workplace safety culture an important factor in patient safety culture, but that they are mutually affected [21, 26, 27]. Both workplace safety culture and patient safety culture are integral to an overall culture of safety and are influenced by overall organizational culture and attitudes toward process improvements, and they are inextricably linked in that improvements in one area influence the other. For example, if providers and staff do not have appropriate equipment or sufficient training to properly use equipment to lift and move patients, patients may fall and providers and staff may also fall or be otherwise injured. Despite this theoretical foundation, there is limited empirical evidence about the crucial relationship between workplace safety culture and patient safety culture. Prior studies have only examined the relationship in single hospitals or hospital units and for a small set of workplace safety culture measures such as workplace violence and burnout [28–30].

This paper presents evidence regarding this crucial gap by analyzing the associations between workplace safety culture and patient safety culture for a large set of patient safety culture and workplace safety culture measures assessed in a diverse set of hospitals with a wide range of characteristics and geographic locations. To perform this analysis, we used data from a pilot test of the AHRQ Surveys on Patient Safety Culture® (SOPS®) Hospital Survey 2.0 Workplace Safety Supplemental Item Set, which was conducted in 28 hospitals across 16 states, which allows for more generalizable findings than data from a single hospital or unit. We hypothesize that more positive workplace safety culture is associated with more positive patient safety culture.

## Methods

### Data sources and measures

We employed a cross-sectional study design which assessed the associations between patient safety culture measures which are the core items from the AHRQ Surveys on Patient Safety Culture® (SOPS®) Hospital Survey 2.0 [31] and workplace safety culture measures from the SOPS Workplace Safety Supplemental Item Set for Hospitals [32]. The SOPS Hospital Survey 2.0, released in 2019, is an update of the original survey released in 2004. Designed to assess hospital provider^2^ and staff^3^ perceptions about patient safety issues and event reporting, the core SOPS Hospital Survey 2.0 includes 32 items aggregated into 10 patient safety culture composite measures and one overall patient safety rating item and one item on the number of events reported (not reported in this study), respectively.

Workplace safety culture is assessed using the Hospital Workplace Safety Supplemental Item Set. This item set was developed by Westat, under contract with AHRQ, in response both to increased concern about healthcare worker safety as a result of the COVID-19 pandemic and a recognition of the importance of workplace safety in ensuring patient safety. The items were developed based on literature on workplace safety in hospitals, interviews with hospital workplace safety experts and researchers, and through feedback from the SOPS Technical Expert Panel (TEP) and workplace safety subject matter experts (SMEs). The development team conducted iterative cognitive testing of the draft survey items with 20 hospital providers and staff and received input from the TEP and SMEs at multiple stages in the development process. The workplace safety supplemental item set includes 16 items aggregated into six composite measures, as well as three single item measures and one overall workplace safety rating.

In 2021, a pilot study was conducted which collected responses to the workplace safety items for 28 hospitals in 16 states across the U.S. The purpose of this pilot study was to obtain data for psychometric analyses to examine the reliability and validity of the Workplace Safety Supplemental Item Set for hospitals. This psychometric analysis of the workplace safety culture measures provided evidence that the measures were reliable and valid [33]. Psychometric analysis of the SOPS Hospital Survey 2.0 have previously shown that the patient safety culture measures are also reliable and valid [34].

Recruitment of hospitals occurred through AHRQ SOPS email listserv subscribers, users of the survey, webinar participants, and through outreach to hospital stakeholder organizations. From the list of interested hospitals, a convenience sample of 28 hospitals were selected that varied by several characteristics (e.g., bed size, region, ownership, teaching status), but were not statistically representative of all U.S. hospitals. The pilot study was a web-based survey administered to a census of all providers and staff in the selected hospitals with the workplace safety items near the end of the survey. Each provider and staff member of the selected hospitals received an email with a unique survey link. At the beginning of the survey, the following statement was included: “The survey is voluntary, but your feedback will help your hospital identify areas for patient safety and workplace safety improvement. If you do not wish to answer a question, you may leave it blank. Westat will keep your individual responses to this survey confidential. Only group results will be reported.”

Out of 19,979 surveys distributed, 7,037 providers and staff responded, resulting in a 35% overall response rate. Across all pilot study hospitals, respondents had the following category of staff position: 35% nurses; 2% physician or physician assistant; 18% other clinical position; 11% management; 20% support, and 13% other staff position [35].

The patient safety measures were as follows, with the number of items in parentheses: Teamwork (3); Staffing and Work Pace (4); Organizational Learning-Continuous Improvement (3); Response to Error (4); Supervisor, Manager, or Clinical Leader Support for Patient Safety (3); Communication About Error (3); Communication Openness (4); Reporting Patient Safety Events (2); Hospital Management Support for Patient Safety (3); Handoffs and Information Exchange (3); and Patient Safety Rating (1) [36].

The workplace safety measures were as follows, with the number of items in parentheses: Protection from Workplace Hazards (3); Moving, Transferring, or Lifting Patients (3); Addressing Workplace Aggression from Patients or Visitors (2); Workplace Aggression Policies, Procedures, and Training (2); Addressing Verbal Aggression From Providers or Staff (1); Supervisor, Manager, or Clinical Leader Support for Workplace Safety (3); Hospital Management Support for Workplace Safety (3); Workplace Safety and Reporting (1); Work Stress/Burnout (1); and Overall Rating on Workplace Safety for Providers and Staff (1) [32].

We calculated hospital-level percent positive scores as the percentage of respondents within a hospital who answered positively (% Strongly agree/Agree or Always/Most of the time) for positively worded items, and (% Strongly disagree/Disagree) for negatively worded items for each item. Percent positive scores can range from 0 to 100. These hospital-level percent positive scores for the items within each composite measure were equally weighted and averaged to compute hospital-level composite measure scores. There was one exception to this scoring: *Work Stress/Burnout* was reported as the percentage of respondents that chose the response ‘3’,^4^ ‘4’,^5^ or ‘5’^6^, indicating they had one or more symptoms of work stress or burnout.

#### Hospital characteristics as Covariates

Three hospital characteristics obtained from the 2020 American Hospital Association (AHA) Annual Survey of Hospitals Database were examined as covariates or control variables. The first control variable was bed size which was categorized into seven categories: 6–24 beds, 25–49 beds, 50–99 beds, 100–199 beds, 200–299 beds, 399 beds, and 400 or more beds. The seven categories were coded as 1 through 7 and this variable was included as a continuous variable modeled linearly in the regression models. The second control variable was ownership status, which was either a government-owned hospital or non-government-owned hospital. The third control variable was teaching status, which was either a teaching or non-teaching hospital. These variables were included because hospital characteristics have been demonstrated to show consistent associations with SOPS Hospital Survey scores [37] and are also likely to be associated with Hospital Workplace Safety Supplemental Item Set measures.

### Analysis sample

All analyses were conducted using the responses of 6,684 providers and staff respondents (353 of the 7,037 respondents did not answer any workplace safety items) from 28 hospitals that participated in the SOPS Hospital Workplace Safety Supplemental Item Set pilot study.

### Analyses

All analyses were conducted using SAS V9 and were at the hospital level.

### Descriptive statistics

Means, standard deviations, and ranges for hospital-level percent positive scores (or percent stress/burnout), were calculated for all workplace safety culture and patient safety culture composite measures, as well as the two workplace safety culture single item measures and the overall ratings for both workplace safety culture and patient safety culture. These descriptive statistics show the variation in the patient safety culture and workplace safety culture measures and provide context for interpreting the regression analyses.

### Multiple regressions

We conducted a series of multiple regressions to examine the associations between hospital workplace safety culture measures and patient safety culture measures. Specifically, each regression model had one patient safety culture measure as the dependent variable, and one workplace safety culture measure as the independent variable along with the control variables (hospital bed size, teaching status, and ownership). To test a key assumption of linear regression, we confirmed that the percent positive (or negative in the case of burnout) values for each measure were normally distributed.

We included only one workplace safety culture measure in each model because tests of variance inflation factors (VIF) showed substantial evidence of multicollinearity when including all workplace safety culture measures in a single regression. Rules-of-thumb for an indication of substantial multicollinearity are VIFs generally between 4 and 10, with a VIF above 10 indicating substantial multicollinearity [38]. Tests of VIF when including all workplace safety culture measures in the same regression indicated a VIF of 12.8 for *Hospital Management Support for Workplace Safety* and a VIF of 12.2 for the *Overall Rating on Workplace Safety for Providers and Staff*.

Because we are simultaneously conducting multiple hypothesis tests, it is important to adjust the *p*-values of the hypothesis tests to control the number of false positives due to chance. We adjusted for multiple hypothesis testing by controlling the false discovery rate which is the expected proportion of false rejections (statistically significant estimates) among all rejected tests, using the standard method by Benjamini and Hochberg [39].

## Results

### Descriptive statistics

Table 1 shows, for each workplace safety culture and patient safety culture measure, the means and standard deviations for all 28 hospitals of the percentage of individual responses that were positive (except for *Work Stress/Burnout*). Percent positive scores for the patient safety culture composite measures ranged from 55.6% (*Staffing and Work Pace*) to 80.6% (*Teamwork)*. The *Patient Safety Rating* percent positive score was 63.9%.

Percent positive scores for the workplace safety composite measures ranged from 58.1% (*Addressing Workplace Aggression From Patients or Visitors*) to 90.3% (*Protection from Workplace Hazards*). *Work Stress/Burnout*, measured as the overall percentage of respondents in a hospital that reported experiencing symptoms of burnout, was 30.4%. The *Overall Rating on Workplace Safety for Providers and Staff* percent positive score was 53.1%. These statistics indicate that while the vast majority of providers and staff report they had adequate physical protection, far fewer reported they had adequate protection from workplace aggression from patients or visitors. Further, substantially fewer providers and staff reported positive ratings of overall workplace safety culture than reported positive ratings of overall patient safety culture.

**Table 1:** Descriptive statistics for patient safety culture and workplace safety culture measures (*N* = 28)

**Patient Safety Culture Measures**				
**Composite Measures**	**Mean Percent Positive Score (%)**	**Standard Deviation (%)**	**Min** **(%)**	**Max** **(%)**
Teamwork	80.6%	5.7%	72.4%	95.1%
Staffing and Work Pace	55.6%	9.7%	38.6%	73.9%
Organizational Learning – Continuous Improvement	68.4%	7.8%	55.0%	84.3%
Response to Error	62.7%	10.1%	41.5%	84.1%
Supervisor, Manager, or Clinical Leader Support for Patient Safety	79.9%	7.5%	61.8%	92.8%
Communication About Error	69.1%	7.6%	57.4%	82.8%
Communication Openness	73.2%	6.9%	62.1%	87.4%
Reporting Patient Safety Events	72.8%	6.1%	62.5%	83.0%
Hospital Management Support for Patient Safety	65.8%	9.9%	50.5%	86.5%
Handoffs and Information Exchange	62.4%	9.8%	44.4%	88.3%
**Overall Rating on Patient Safety**	**Mean Percent Positive Score**	**Standard Deviation**	**Min** **(%)**	**Max** **(%)**
Patient Safety Rating	63.9%	10.7%	40.7%	84.2%
**Workplace Safety Culture Measures**				
**Composite Measures**	**Mean Percent Positive Score (%)**	**Standard Deviation (%)**	**Min** **(%)**	**Max** **(%)**
Protection From Workplace Hazards	90.3%	4.4%	80.0%	98.1%
Moving, Transferring, or Lifting Patients	73.5%	9.9%	49.2%	88.7%
Addressing Workplace Aggression From Patients or Visitors	58.1%	12.7%	33.5%	89.4%
Workplace Aggression, Policies, Procedures, and Training	68.5%	10.8%	44.5%	83.3%
Supervisor, Manager, or Clinical Leader Support for Workplace Safety	81.6%	6.9%	62.8%	93.3%
Hospital Management Support for Workplace Safety	70.5%	10.2%	54.8%	87.8%
**Single Item Measures**	**Mean Percent Positive Score (%)**	**Standard Deviation (%)**	**Min** **(%)**	**Max** **(%)**
Addressing Verbal Aggression From Providers or Staff	78.4%	9.0%	63.6%	100%
Workplace Safety and Reporting	78.3%	8.1%	58.3%	92.1%
	**Mean Percent Experiencing Symptoms of Work Stress/ Burnout (%)**	**Standard Deviation**	**Min** **(%)**	**Max** **(%)**
Work Stress/Burnout	30.4%	8.5%	13.6%	55%
**Overall Rating on Workplace Safety**	**Mean Percent Positive Score (%)**	**Standard Deviation (%)**	**Min** **(%)**	**Max** **(%)**
Overall Rating on Workplace Safety for Providers and Staff	53.1%	11.1%	35.0%	76.7%

### Multiple regressions

Tables S1a and S1b present the results of multiple linear regressions examining associations for workplace safety culture measures with the patient safety culture measures. Table S1b includes the number of statistically significant associations and the mean and range of the standardized regression coefficients of those statistically significant associations for each workplace safety culture measure. Of the 110 regression estimates, 69 were statistically significant (*p* < 0.05). Tables S2a and S2b provide model fit statistics of each of the regression models.

Three workplace safety culture measures were significantly associated with all 11 patient safety culture measures and had the largest average magnitude associations (*Overall Rating on Workplace Safety for Providers and Staff*, mean β = 0.67; *Supervisor, Manager, or Clinical Leader Support for Workplace Safety*, mean β = 0.62; and *Hospital Management Support for Workplace Safety*, mean β = 0.62). These three measures had the largest number of associations with patient safety culture measures on average and represented four of the five largest magnitude associations with patient safety culture measures: *Overall Rating on Workplace Safety for Providers and Staff* and *Hospital Management Support for Patient Safety* (β = 0.95); *Hospital Management Support for Workplace Safety* and *Hospital Management Support for Patient Safety (*β = 0.93); *Supervisor, Manager, or Clinical Leader for Workplace Safety* and *Response to Error* (β = 0.90); and, *Overall Rating on Workplace Safety for Providers and Staff* and *Overall Patient Safety Rating (*β = 0.85).

Two workplace safety culture measures (*Protection from Workplace Hazards*, mean β = 0.57 and *Workplace Safety and Reporting*, mean β = 0.53) were significantly associated with 10 of the 11 patient safety culture measures. Associations with *Protection from Workplace Hazards* ranged from 0.39 with *Reporting Patient Safety Events* to 0.79 with *Hospital Management Support for Patient Safety*. Associations with *Workplace Safety and Reporting* ranged from 0.28 with *Reporting Patient Safety Events* to 0.75 with *Response to Error*.

Two workplace safety culture measures (*Moving, Transferring, or Lifting Patients* and *Work Stress/Burnout*) were significantly associated with seven out of 11 patient safety culture measures. Statistically significant associations of *Moving, Transferring, or Lifting Patients* with patient safety culture measures had an average of β = 0.57, ranging from 0.31 with *Reporting Patient Safety Events* to 0.87 with *Hospital Management Support for Patient Safety*. Statistically significant associations of *Work Stress/Burnout* with patient safety culture measures had an average of β = -0.53, ranging from − 0.47 with *Organizational Learning – Continuous Improvement* to -0.60 with *Staffing and Work Pace*. Associations were negative, indicating that higher *Work Stress/Burnout* was associated with lower patient safety culture.

The three workplace aggression measures (*Addressing Workplace Aggression from Patients or Visitors*; *Workplace Aggression Policies, Procedures, and Training*; and *Addressing Verbal Aggression from Providers or Staff*) had the lowest number of significant associations and smallest associations on average, with two or fewer significant relationships per measure with the patient safety culture measures. Specifically, *Addressing Workplace Aggression from Patients or Visitors* was significantly associated with only *Communication Openness* (β = 0.42); *Workplace Aggression Policies, Procedures, and Training* was not significantly associated with any patient safety culture measures; and *Addressing Verbal Aggression from Providers or Staff* was significantly associated with two patient safety culture measures (mean β = 0.56, ranging from 0.50 with *Response to Error* to 0.61 with *Teamwork*).

## Discussion

We examined the relationship between hospital provider and staff perceptions of workplace safety culture and patient safety culture. Our analyses revealed 69 out of 110 statistically significant associations between the workplace safety and patient safety culture measures, while controlling for hospital bed size, ownership, and teaching status, and controlling for multiple comparisons. All workplace safety measures were significantly associated with at least half of the patient safety culture measures, except for the three measures related to addressing workplace aggression from patients or other staff; these measures were only associated with up to two patient safety culture measures.

Theoretical models of organizational culture in health care have posited that the values and strategy of leadership along with characteristics of organizational structure and culture heavily influence the intermediate process domains of staffing; training; employee safety through protection from workplace hazards; resources to safely care for patients and themselves including proper equipment and staffing to move and lift patients safely; and other factors [27]. These process domains play a key role in how well providers and staff collaborate and are focused on patients and their safety, which in turn influences both satisfaction and intention to leave of providers and staff as well as patient satisfaction and clinical outcomes [30]. This study provides empirical evidence to support multiple aspects of this model. In particular, hospital management support for and an overall perception of a healthy and robust workplace safety culture have the strongest associations with perceptions of patient safety culture. Additionally, feeling free to report workplace safety incidents without negative consequences, having sufficient resources to protect themselves from hazards, and being able to move and lift patients safely are also strongly associated with staff and providers’ perceptions of patient safety culture.

The strongest association with *Work Stress/Burnout* was with *Staffing and Work Pace*, which provides evidence that lower stress and burnout of providers and staff is associated with having sufficient staff, reasonable working hours, and better work pace. The strong relationships between higher burnout and poor patient safety culture are consistent with prior literature [29, 30, 40–43].

The three measures regarding workplace aggression (policies, procedures, and training; and addressing workplace aggression from patients or visitors and other providers or staff) were not as strongly associated with the patient safety culture measures as the other workplace safety culture measures. We performed a detailed investigation to explore these results and found that two outlier hospitals were the primary reason for the relatively large negative (though nonsignificant) associations between the *Workplace Aggression Policies, Procedures, and Training* composite measure and the patient safety culture measures. However, these outliers do not explain the low magnitudes and sometimes negative direction of the remaining associations between the workplace aggression and patient safety culture measures. Further research is required to assess why associations between the aggression measures and patient safety culture measures may be smaller or whether these results are limited to this particular sample.

This study has several limitations. First, while the number of hospitals is relatively large among the empirical literature on the relationship between patient safety and workplace safety cultures, the number of hospitals is still relatively small. Second, even though the study hospitals were diverse on a number of characteristics, they were selected as a convenience sample and thus are not representative of all U.S. hospitals. Third, the study is cross-sectional and examines associations, so we were unable to provide evidence on how changes in measures vary with changes in other measures or attribute causal directions to the relationships. That is, although workplace safety culture measures were used as the independent variables in the model, we cannot say definitively that better workplace safety causes better patient safety culture, but only that they are related and likely influence each other.

## Conclusions

The analyses presented in this paper revealed relationships between patient safety culture and workplace safety culture measures. We found statistically significant associations between the majority of the workplace safety culture and patient safety culture measures, confirming our hypothesis that these important perceptions would be positively related. Overall, support from hospital management and supervisors, manager, or clinical leaders to ensure workplace safety, being able to report safety problems without negative consequences, and the overall rating of workplace safety culture were the workplace safety culture measures most strongly associated with patient safety culture.

These results provide empirical evidence to support the contention that the concepts of workplace safety culture and patient safety culture are fundamentally linked, and both are integral to a strong and healthy culture of safety. Future research should focus on collecting additional evidence about this relationship using larger sample sizes and additional measures to substantiate these results. This relationship could be assessed outside of hospital settings; nursing homes, for example, could provide fertile ground for additional research, given AHRQ’s recent release of a SOPS Workplace Safety Supplemental Item Set for Nursing Homes. Finally, the relationship between measures of aggression and patient safety culture should be further studied conceptually and empirically to determine whether the weak relationship presented in this study is generalizable to other U.S. hospitals.

### Electronic supplementary material

Below is the link to the electronic supplementary material.

Supplementary Material 1

Supplementary Material 2

## Data Availability

Some of the de-identified SOPS Hospital Survey 2.0 data are available upon request for research purposes.

## Footnotes

1 An adverse event in healthcare is also known as a “patient safety event” which is defined differently by different government agencies and healthcare organizations. On the Surveys on Patient Safety Culture® (SOPS®), a “patient safety event” is defined as “any type of healthcare-related error, mistake, or incident, regardless of whether or not it results in patient harm.”

2 Provider refers to physicians, physician assistants, and nurse practitioners who diagnose, treat patients, and prescribe medications.

3 Staff refers to all other individuals who work in the hospital but are not providers. Examples include medical assistants, administrative staff, housekeeping, and nutrition.

4 Response option 3 for the *Work Stress/Burnout* item is: “I am beginning to burn out and have one or more symptoms of burnout, e.g.; emotional exhaustion.”

5 Response option 4 for the *Work Stress/Burnout* item is: “The symptoms of burnout I am experiencing won’t go away. I think about work frustrations a lot.”

6 Response option 5 for the *Work Stress/Burnout* item is: “I feel completely burned out. I am at the point where I may need to seek help.”
