# Supplementary material for: Associations between patient safety culture and workplace safety culture in hospital settings
Source: BMC Health Serv Res. 2024 May 2;24:568. doi: 10.1186/s12913-024-10984-3 (PMC11065685; doi:10.1186/s12913-024-10984-3)
Supplement: Supplementary file 1 — Supplementary Material 1 [file 12913_2024_10984_MOESM1_ESM.docx]

**Table S1a. Multiple Linear Regression Results for Associations Between Workplace Safety Culture and Patient Safety Culture Measures – Part 1**

| **Patient Safety Culture Measures** | **Teamwork** | **Staffing and Work Pace** | **Organizational Learning – Continuous Improvement** | **Response to Error** | **Supervisor, Manager, or Clinical Leader Support for Patient Safety** | **Communication About Error** |
| --- | --- | --- | --- | --- | --- | --- |
| **Workplace Safety Composite Measures** | | | | | | |
| Protection from Workplace Hazards | 0.36 | 0.40* | 0.62* | 0.60* | 0.51* | 0.60* |
| Moving, Transferring, or Lifting Patients | 0.32 | 0.17 | 0.59* | 0.54* | 0.21 | 0.62* |
| Addressing Workplace Aggression from Patients or Visitors | 0.23 | 0.08 | 0.25 | 0.43 | 0.13 | 0.47 |
| Aggression Policies, Procedures, and Training | -0.03 | -0.25 | 0.05 | -0.04 | -0.18 | -0.18 |
| Supervisor, Manager, or Clinical Leader Support for Workplace Safety | 0.48* | 0.48* | 0.81* | 0.90* | 0.83* | 0.81* |
| Hospital Management Support for Workplace Safety | 0.44* | 0.43* | 0.76* | 0.76* | 0.42* | 0.74* |
| **Workplace Safety Single Item Measures** | | | | | | |
| Addressing Verbal Aggression from Providers and Staff | 0.61* | 0.06 | 0.43 | 0.50* | 0.2 | 0.41 |
| Workplace Safety and Reporting | 0.47* | 0.39* | 0.59* | 0.75* | 0.50* | 0.63* |
| Work Stress/ Burnout^1^ | -0.31 | -0.60* | -0.47* | -0.52* | -0.56* | -0.48* |
| **Workplace Safety Overall Rating** | | | | | | |
| Overall Rating on Workplace safety | 0.41* | 0.51* | 0.83* | 0.78* | 0.53* | 0.80* |

Notes: All estimates are standardized regression coefficients, in standard deviation units with absolute values ranging from 0 to 1. Models control for bed size, ownership, and teaching status. Models control for multiple hypothesis testing through controlling the family-wise error rate (FWER) using the Benjamini-Hochberg procedure. *p<0.05.

^1^ Higher scores represent more work stress/burnout.

**Table S1b. Multiple Linear Regression Results for Associations Between Workplace Safety Culture and Patient Safety Culture Measures – Part 2**

| **Patient Safety Culture Measures** | **Communication Openness** | **Reporting Patient Safety Events** | **Hospital Management Support for Patient Safety** | **Handoffs and Information Exchange** | **Overall Patient Safety Rating** | **Mean (and Range) of Standardized Coefficients for Significant Relationships** | **Total Number of Significant Relationships**  **(Tables S1a and S1b)** |
| --- | --- | --- | --- | --- | --- | --- | --- |
| **Workplace Safety Composite Measures** | | | | | | | |
| Protection from Workplace Hazards | 0.59* | 0.39* | 0.79* | 0.58* | 0.65* | **0.57**  **(0.39-0.79)** | **10** |
| Moving, Transferring, or Lifting Patients | 0.51* | 0.31* | 0.87* | 0.34 | 0.56* | **0.57**  **(0.31-0.87)** | **7** |
| Addressing Workplace Aggression from Patients or Visitors | 0.42* | 0.02 | 0.42 | 0.27 | 0.27 | **0.42**  **N/A^1^** | **1** |
| Workplace Aggression Policies, Procedures, and Training | -0.21 | -0.14 | 0.08 | -0.41 | -0.09 | **N/A^2^** | **0** |
| Supervisor, Manager, or Clinical Leader Support for Workplace Safety | 0.57* | 0.38* | 0.62* | 0.42* | 0.56* | **0.62**  **(0.38-0.90)** | **11** |
| Hospital Management Support for Workplace Safety | 0.69* | 0.38* | 0.93* | 0.54* | 0.75* | **0.62**  **(0.38-0.93)** | **11** |
| **Workplace Safety Single Item Measures** | | | | | | | |
| Addressing Verbal Aggression from Providers and Staff | 0.28 | -0.01 | 0.41 | 0.15 | 0.23 | **0.56**  **(0.50-0.61)** | **2** |
| Workplace Safety and Reporting | 0.52* | 0.28* | 0.57* | 0.39 | 0.56* | **0.53**  **(0.28-0.75)** | **10** |
| Work Stress/ Burnout^3^ | -0.45 | -0.28 | -0.35 | -0.48 | -0.58* | **-0.54**  **(-0.60—0.47)** | **6** |
| **Workplace Safety Overall Rating** | | | | | | | |
| Overall Rating on Workplace safety | 0.73* | 0.45* | 0.95* | 0.57* | 0.85* | **0.67**  **(0.41-0.95)** | **11** |

Notes: All estimates are standardized regression coefficients, in standard deviation units with absolute values ranging from 0 to 1. Models control for bed size, ownership, and teaching status. Models control for multiple hypothesis testing through controlling the family-wise error rate (FWER) using the Benjamini-Hochberg procedure. *p<0.05.

^1^ The range of the standardized coefficients for significant relationships could not be calculated because there was only 1 significant relationship.

^2^ The mean and range of the standardized coefficients for significant relationships could not be calculated because there were 0 significant relationships.

^3^ Higher scores represent more work stress/burnout.
