# Supplementary material for: Associations between patient safety culture and workplace safety culture in hospital settings
Source: BMC Health Serv Res. 2024 May 2;24:568. doi: 10.1186/s12913-024-10984-3 (PMC11065685; doi:10.1186/s12913-024-10984-3)
Supplement: Supplementary file 2 — Supplementary Material 2 [file 12913_2024_10984_MOESM2_ESM.docx]

**Table S2a. Model Fit Statistics for Associations Between Workplace Safety Culture and Patient Safety Culture Measures – Part 1**

| **Patient Safety Culture Measures** | **Teamwork** | **Staffing and Work Pace** | **Organizational Learning – Continuous Improvement** | **Response to Error** | **Supervisor, Manager, or Clinical Leader Support for Patient Safety** | **Communication About Error** |
| --- | --- | --- | --- | --- | --- | --- |
| **Workplace Safety Composite Measures** | | | | | | |
| Protection from Workplace Hazards | 0.44 [0.05] | 0.46 [0.08] | 0.60 [0.05] | 0.61 [0.07] | 0.52 [0.06] | 0.56 [0.06] |
| Moving, Transferring, or Lifting Patients | 0.40 [0.05] | 0.34 [0.09] | 0.49 [0.06] | 0.48 [0.08] | 0.32 [0.07] | 0.50 [0.06] |
| Addressing Workplace Aggression from Patients or Visitors | 0.36 [0.05] | 0.32 [0.09] | 0.29 [0.07] | 0.40 [0.08] | 0.30 [0.07] | 0.39 [0.06] |
| Aggression Policies, Procedures, and Training | 0.32 [0.05] | 0.38 [0.08] | 0.25 [0.07] | 0.27 [0.09] | 0.32 [0.07] | 0.26 [0.07] |
| Supervisor, Manager, or Clinical Leader Support for Workplace Safety | 0.49 [0.04] | 0.48 [0.08] | 0.70 [0.05] | 0.83 [0.04] | 0.78 [0.04] | 0.69 [0.05] |
| Hospital Management Support for Workplace Safety | 0.48 [0.04] | 0.47 [0.08] | 0.74 [0.04] | 0.76 [0.05] | 0.44 [0.06] | 0.70 [0.05] |
| **Workplace Safety Single Item Measures** | | | | | | |
| Addressing Verbal Aggression from Providers and Staff | 0.53 [0.04] | 0.32 [0.09] | 0.35 [0.07] | 0.41 [0.08] | 0.31 [0.07] | 0.32 [0.07] |
| Workplace Safety and Reporting | 0.50 [0.04] | 0.44 [0.08] | 0.52 [0.06] | 0.71 [0.06] | 0.49 [0.06] | 0.54 [0.06] |
| Work Stress/ Burnout^1^ | 0.41 [0.05] | 0.65 [0.06] | 0.45 [0.06] | 0.52 [0.08] | 0.58 [0.05] | 0.45 [0.06] |
| **Workplace Safety Overall Rating** | | | | | | |
| Overall Rating on Workplace safety | 0.45 [0.05] | 0.51 [0.07] | 0.75 [0.04] | 0.71 [0.06] | 0.49 [0.06] | 0.70 [0.05] |

Notes: Numbers not in brackets are R-squared values and numbers in brackets are the root mean squared error of multiple regression models with the measure in the column as the dependent variable and the measure in the row as the independent variable. Models also control for bed size, ownership, and teaching status.

^1^Higher scores represent more work stress/burnout.

**Table S2b. Model Fit Statistics for Associations Between Workplace Safety Culture and Patient Safety Culture Measures – Part 2**

| **Patient Safety Culture Measures** | **Communication Openness** | **Reporting Patient Safety Events** | **Hospital Management Support for Patient Safety** | **Handoffs and Information Exchange** | **Overall Patient Safety Rating** |
| --- | --- | --- | --- | --- | --- |
| **Workplace Safety Composite Measures** |  |  |  |  |  |
| Protection from Workplace Hazards | 0.58 [0.05] | 0.61 [0.04] | 0.68 [0.06] | 0.44 [0.08] | 0.61 [0.07] |
| Moving, Transferring, or Lifting Patients | 0.44 [0.06] | 0.54 [0.04] | 0.65 [0.06] | 0.21 [0.09] | 0.45 [0.09] |
| Addressing Workplace Aggression from Patients or Visitors | 0.39 [0.06] | 0.47 [0.05] | 0.24 [0.09] | 0.18 [0.10] | 0.28 [0.10] |
| Workplace Aggression Policies, Procedures, and Training | 0.30 [0.06] | 0.49 [0.05] | 0.12 [0.10] | 0.29 [0.09] | 0.24 [0.10] |
| Supervisor, Manager, or Clinical Leader Support for Workplace Safety | 0.49 [0.05] | 0.57 [0.04] | 0.38 [0.08] | 0.25 [0.09] | 0.45 [0.09] |
| Hospital Management Support for Workplace Safety | 0.67 [0.04] | 0.59 [0.04] | 0.86 [0.04] | 0.38 [0.08] | 0.71 [0.06] |
| **Workplace Safety Single Item Measures** |  |  |  |  |  |
| Addressing Verbal Aggression from Providers and Staff | 0.30 [0.06] | 0.47 [0.05] | 0.21 [0.10] | 0.14 [0.10] | 0.26 [0.10] |
| Workplace Safety and Reporting | 0.47 [0.05] | 0.53 [0.05] | 0.37 [0.09] | 0.25 [0.09] | 0.48 [0.08] |
| Work Stress/ Burnout^1^ | 0.45 [0.06] | 0.54 [0.04] | 0.23 [0.09] | 0.34 [0.09] | 0.54 [0.08] |
| **Workplace Safety Overall Rating** |  |  |  |  |  |
| Overall Rating on Workplace safety | 0.65 [0.04] | 0.62 [0.04] | 0.78 [0.05] | 0.36 [0.08] | 0.77 [0.06] |

Notes: Numbers not in brackets are R-squared values and numbers in brackets are the root mean squared error of multiple regression models with the measure in the column as the dependent variable and the measure in the row as the independent variable. Models also control for bed size, ownership, and teaching status.

^1^Higher scores represent more work stress/burnout.
